# Protective Effects of the Phytocystatin CaneCPI‑5 on Periodontal Ligament Cell Osteogenic Activity and Alveolar Bone Loss under Inflammatory Challenge

**DOI:** 10.1021/acsptsci.6c00363

**Published:** 2026-09-30

**Authors:** Maria Eduarda Scordamaia Lopes, Angélica Letícia Reis Pavanelli, Sâmmea Martins Vieira, Gisele Faria, Flávio Henrique Silva, Sigrun Eick, Andressa V. B. Nogueira, James Deschner, Joni Augusto Cirelli

**Affiliations:** † School of Dentistry at Araraquara, Department of Diagnosis and Surgery, Sao Paulo State University (UNESP), Araraquara 14801-903, Brazil; ‡ School of Dentistry at Araraquara, Department of Restorative Dentistry, Sao Paulo State University (UNESP), Araraquara 14801-903, Brazil; § Department of Genetics and Evolution, Federal University of São Carlos (UFSCAR), São Carlos 13565-905, Brazil; ∥ Laboratory of Oral Microbiology, Department of Periodontology, 27210 University of Bern, Bern 3012, Switzerland; ⊥ Department of Periodontology and Operative Dentistry, 39068 University Medical Center of the Johannes Gutenberg University, Mainz 55131, Germany; # Research Center for Immunotherapy (FZI), University Medical Center of the Johannes Gutenberg University, Mainz 55131, Germany

**Keywords:** periodontitis, cystatins, periodontal ligament cells, osteogenic differentiation, alveolar bone loss

## Abstract

Periodontal disease is characterized by a chronic inflammatory environment that compromises the regenerative capacity of periodontal tissues, particularly by impairing the osteogenic potential of periodontal ligament cells. Phytocystatins have emerged as bioactive molecules with potential regenerative and immunomodulatory properties; however, their effects on periodontal tissues under inflammatory conditions remain unexplored. In this study, we investigated the effects of CaneCPI-5 on the osteogenic behavior of human periodontal ligament cells (hPDLCs) under basal and inflammatory conditions in vitro, as well as its impact on alveolar bone preservation and periodontal tissue responses in a murine model of experimental periodontitis. In vitro, CaneCPI-5 promoted the expression of osteogenic markers and supported mineralized matrix formation under basal conditions. Notably, CaneCPI-5 preserved osteogenic gene expression and mineral deposition in hPDLCs exposed to *Filifactor alocis*, indicating a sustained osteogenic response despite inflammatory stimulation. In vivo, treatment with CaneCPI-5 significantly attenuated alveolar bone loss, increased bone mineral density and bone volume fraction, and was associated with histological features indicative of a more preserved periodontal tissue architecture compared with untreated disease controls. Stereometric analyses further revealed reduced inflammatory cell infiltration and vascular density, alongside increased fibroblast presence and extracellular matrix components in treated animals. Collectively, these findings demonstrate that CaneCPI-5 supports osteogenic activity and contributes to periodontal tissue preservation in inflammatory settings. The convergence of in vitro and in vivo data suggests that CaneCPI-5 may be a promising bioactive molecule for the development of adjunctive regenerative strategies to mitigate inflammation-associated periodontal tissue destruction.

## Graphical abstract

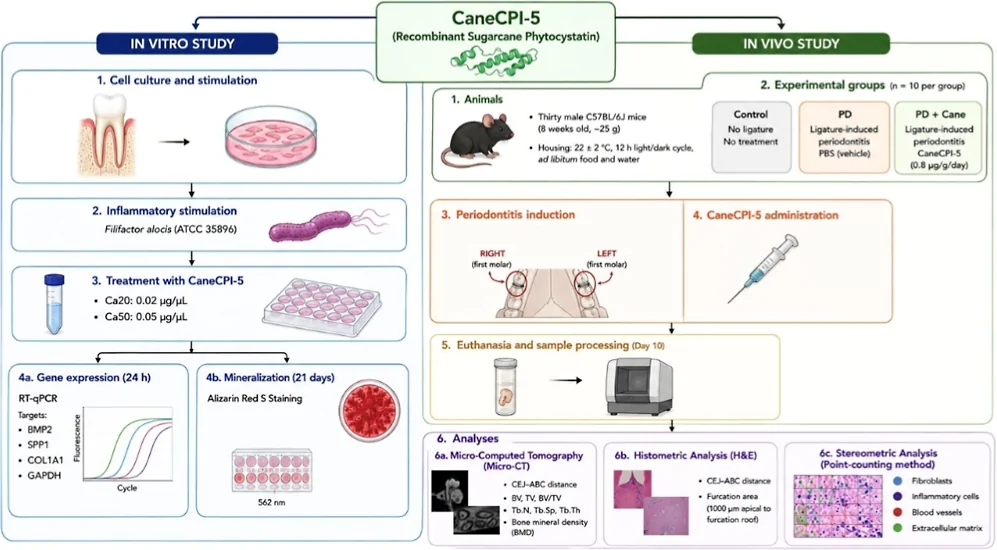

Periodontal disease is characterized by a persistent inflammatory process resulting from an imbalance in the microbial ecosystem of the dental biofilm, leading to progressive damage to the supporting tissues of the teeth. This destructive process compromises the integrity of the periodontal ligament, alveolar bone, and cementum, ultimately impairing oral function and potentially resulting in tooth loss.^1–3^ Given its high prevalence among adult populations and its cumulative impact on oral health, periodontitis remains a major challenge in both clinical practice and public health.^4,5^ Importantly, tissue breakdown in periodontal disease is not solely driven by microbial presence, but largely by an exacerbated host inflammatory response that disrupts tissue homeostasis and limits endogenous repair mechanisms.^6^ These considerations underscore the need for therapeutic approaches that promote tissue regeneration in addition to controlling infection.

The periodontal ligament represents a dynamic connective tissue with a central role in maintaining alveolar bone integrity.^7,8^ Cells derived from this tissue exhibit osteogenic potential and contribute to extracellular matrix synthesis, positioning them as key players in periodontal regeneration.^9,10^ However, the inflammatory microenvironment typical of periodontal disease profoundly alters the biological behavior of periodontal ligament cells, leading to impaired osteogenic differentiation and reduced mineralized matrix formation.^9^ This inflammatory-mediated dysfunction highlights the importance of evaluating candidate regenerative agents not only under physiological conditions but also in environments that more closely resemble the pathological context of periodontitis.^11^

In recent years, naturally derived bioactive molecules have emerged as promising modulators of tissue repair and regeneration.^12^ Among these, phytocystatins—plant-derived cysteine protease inhibitors—have demonstrated diverse biological activities related to cellular regulation and tissue remodeling.^13–15^ In this context, bone remodeling is tightly regulated by the balance between osteoblastic bone formation and osteoclastic bone resorption, processes in which cysteine proteases, particularly cathepsins, play essential roles.^16,17^ Among them, cathepsin K is considered a key mediator of bone matrix degradation and has been recognized as an important therapeutic target in bone loss disorders.^18^ Consequently, cystatins, natural inhibitors of cysteine proteases, have attracted increasing interest due to their potential to modulate bone metabolism and inflammatory tissue destruction. Previous studies have demonstrated that CaneCPI-5, a phytocystatin isolated from sugar cane, modulates biological processes associated with tissue homeostasis, including altering oral biofilm development and stimulating osteoblast adhesion, migration, proliferation, and differentiation.^19,20^ These findings suggest that CaneCPI-5 may influence both inflammatory and regenerative pathways relevant to periodontal tissue preservation.

Based on these observations, the present study aimed to investigate whether CaneCPI-5 can preserve the osteogenic behavior of human periodontal ligament cells under inflammatory conditions in vitro and whether these effects are associated with protection against periodontal tissue breakdown and alveolar bone loss in a murine model of experimental periodontitis.

## Results and Discussion

### CaneCPI-5 Preserves Osteogenic Activity of hPDLCs under Basal and Inflammatory Conditions

The present study demonstrates that CaneCPI-5 exerts a protective effect on periodontal ligament cell osteogenic activity under inflammatory conditions, a critical challenge in the context of periodontal disease. Beyond confirming its pro-osteogenic effects under basal conditions, our findings show that CaneCPI-5 preserves osteogenic gene expression and functional mineralization even in the presence of a bacterial stimulus such as *Filifactor alocis*, a periodontal pathogen associated with disease progression.^21–23^ This ability to sustain osteogenic potential under inflammatory stress is particularly relevant, as, in periodontitis, periodontal tissues are chronically exposed to microbial challenges that disrupt regenerative signaling pathways.^6,11^

RT-qPCR analysis demonstrated that CaneCPI-5, at both tested concentrations, exerted a pro-osteogenic effect under basal conditions, as evidenced by increased expression of osteogenesis-related genes (Figure 1A-C). To further evaluate its activity in an inflammatory environment resembling that observed in periodontal disease, cells were stimulated with *F. alocis*. Compared with the control group, *F. alocis* stimulation significantly reduced SPP1 expression, whereas no significant differences were observed for BMP2 or COL1A1 expression or mineralized matrix formation. Under these inflammatory conditions, CaneCPI-5 at the higher concentration (0.05 μg/μL) maintained its pro-osteogenic effect, with elevated expression levels of the analyzed osteogenic markers (Figure 1D–F). Consistently, the assessment of mineralized matrix formation revealed that CaneCPI-5 at the higher concentration stood out by sustaining mineral deposition even under inflammatory stimulation, compared with the other experimental groups (Figure 1G–H). An important aspect of the present in vitro model is that osteogenic activity was evaluated under conventional culture conditions rather than using an exogenous osteogenic differentiation medium. Thus, the effects observed following CaneCPI-5 treatment were not dependent on the addition of classical osteoinductive supplements, suggesting that CaneCPI-5 can promote osteogenic responses independently of an externally induced osteogenic differentiation program. Nevertheless, these findings should be interpreted within the limitations of an in vitro model, which cannot fully reproduce the complex periodontal microenvironment in vivo.

**Figure 1:**
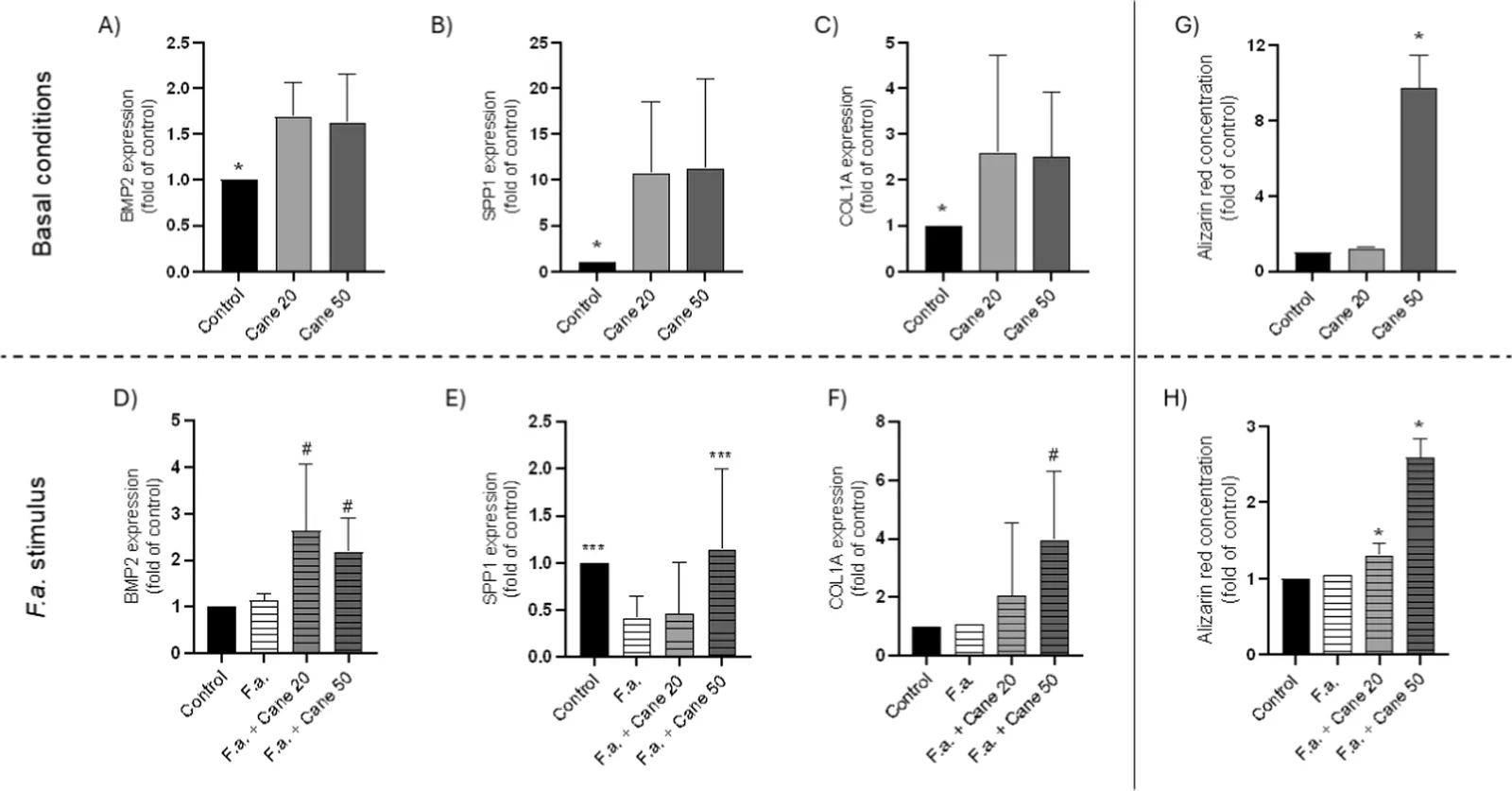
A–H)—Mean and standard deviation of BMP2, SPP1, and COL1A gene expression in hPDLCs under basal conditions (A–C) and following inflammatory stimulation with *F* (D–F). Alizarin Red quantification shows the mean and standard deviation of mineralized matrix formation under basal conditions (G) and *F. alocis* stimulation (H). Cells were treated with CaneCPI-5 at 0.02 μg/μL (Cane 20) or 0.05 μg/μL (Cane 50), alone or in combination with *F. alocis*. Data are expressed as fold change relative to the control group (ANOVA/Tukey, *p* < 0.05). *Significant difference compared with all other groups. #Significant difference compared with Control and F.a. Groups. ***Significant difference compared with *F.a.* Group.

Phytocystatins, including CaneCPI-5 and cystatins derived from Citrus species, have been reported to modulate inflammatory and proteolytic processes by inhibiting cysteine proteases involved in tissue degradation and immune dysregulation. The osteogenic effects observed in the present study are supported by previous mechanistic investigations demonstrating that CaneCPI-5 directly promotes osteoblast differentiation. In preosteoblastic cells, CaneCPI-5 increased the expression of key osteogenic regulators, including BMP2, BMP7, Osterix, and alkaline phosphatase, all of which are critically involved in osteogenic commitment and maturation.^20^ Beyond its direct effects on osteogenic differentiation, CaneCPI-5 has been shown to downregulate inflammatory mediators while promoting angiogenesis and collagen deposition in vivo, suggesting that its biological activity extends to multiple processes involved in tissue repair and regeneration.^15^ Similarly, Citrus-derived phytocystatins such as CsinCPI-2 have demonstrated both anti-inflammatory and pro-osteogenic effects, including enhancement of alkaline phosphatase activity and mineralized nodule formation, as well as attenuation of alveolar bone loss in preclinical models of periodontal disease.^13,14^ In this context, the present in vitro results may be consistent with previously described biological activities of phytocystatins, including the modulation of protease activity and inflammatory responses. Although this study did not directly evaluate protease inhibition or inflammatory signaling pathways, these mechanisms represent plausible explanations that warrant further investigation. Another potential mechanism involves the activation of ERK signaling. Previous studies demonstrated that CaneCPI-5 increases ERK phosphorylation during osteoblast differentiation, a pathway recognized as a critical regulator of osteogenic gene expression, matrix maturation, and mineralization.^20^ While ERK activation was not evaluated in the present study, it is conceivable that maintenance of osteogenic activity in hPDLCs exposed to inflammatory stimulation may partially depend on similar signaling events. Future studies should investigate cytokine and chemokine profiles, osteoclast differentiation and activity, cysteine protease and cathepsin activity, as well as relevant intracellular signaling pathways. Such investigations will be important to establish mechanistic links between CaneCPI-5 treatment and the preservation of periodontal tissue homeostasis and to further support its translational potential.

Inflammatory microenvironments characteristic of periodontitis have been shown to impair the osteogenic potential of periodontal ligament cells. Inflammatory stimuli, including bacterial virulence factors and cytokines, can disrupt osteogenic differentiation pathways in PDLCs, resulting in reduced mineralization capacity and altered expression of key osteogenic markers.^8,9,24,25^ The ability of CaneCPI-5 to maintain osteogenic gene expression and functional mineral deposition in the presence of an inflammatory challenge may therefore be relevant to periodontal regeneration by counteracting inflammation-induced impairment of PDLC function. Moreover, mineralized matrix formation represents a functional indicator of osteogenic activity, reflecting the cumulative outcome of multiple transcriptional and signaling pathways.^26^ *F. alocis* was selected as a defined bacterial stimulus based on its recognized association with periodontitis and our previous characterization of this species as a reproducible bacterial challenge in hPDLCs.^27–29^ Nevertheless, although this experimental approach provided a controlled and reproducible bacterial stimulus, the in vitro inflammatory challenge was performed using a single periodontal bacterial species, *F. alocis*, which does not reproduce the polymicrobial nature of the periodontal microbiome. Future studies using multispecies bacterial consortia or more complex microbial communities will therefore be necessary to determine whether the effects of CaneCPI-5 observed here are maintained under conditions that more closely resemble the microbial complexity of periodontal disease.

### CaneCPI-5 Reduces Alveolar Bone Loss and Modulates Periodontal Tissue Response

The in vivo findings further reinforce the translational relevance of CaneCPI-5. In the ligature-induced periodontitis model, treatment with CaneCPI-5 attenuated alveolar bone loss and preserved bone microstructural parameters, outcomes consistent with a protective effect in an inflammatory periodontal environment. While this model does not fully replicate the complexity of human periodontitis, it is well established for evaluating host responses to therapeutic interventions.^30^ Histometric analysis demonstrated that animals treated with CaneCPI-5 exhibited significantly reduced linear alveolar bone loss and furcation area bone loss compared with the PD group (Figure 2A,B). In addition, stereometric evaluation revealed a lower number of inflammatory cells and blood vessels, accompanied by an increased presence of fibroblasts and extracellular matrix components in the CaneCPI-5-treated group (Figure 2C). These findings are consistent with the micro-CT analysis, which showed higher BMD, increased BV/TV, and reduced linear alveolar bone loss in animals receiving CaneCPI-5 treatment (Figure 3).

**Figure 2:**
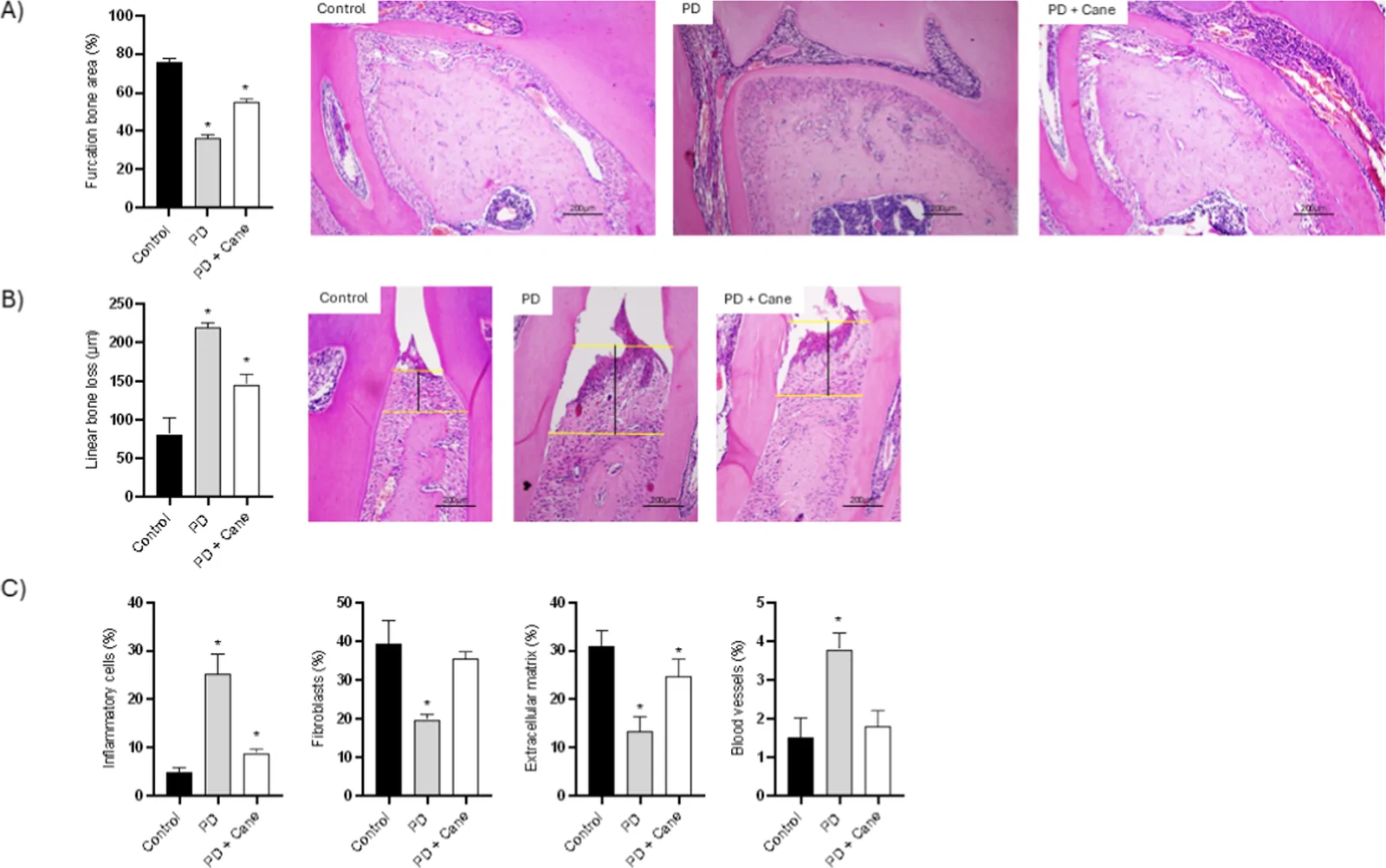
(A–C)—Mean and SD of furcation bone area (A) and linear bone loss (B) measured by histomorphometric analysis. Representative photomicrographs illustrating histological sections of each group. (C) Mean and SD percentage of inflammatory cells, fibroblasts, extracellular matrix and blood vessels. *Significant difference compared with all other groups. (ANOVA/Tukey, *p* < 0.05).

**Figure 3:**
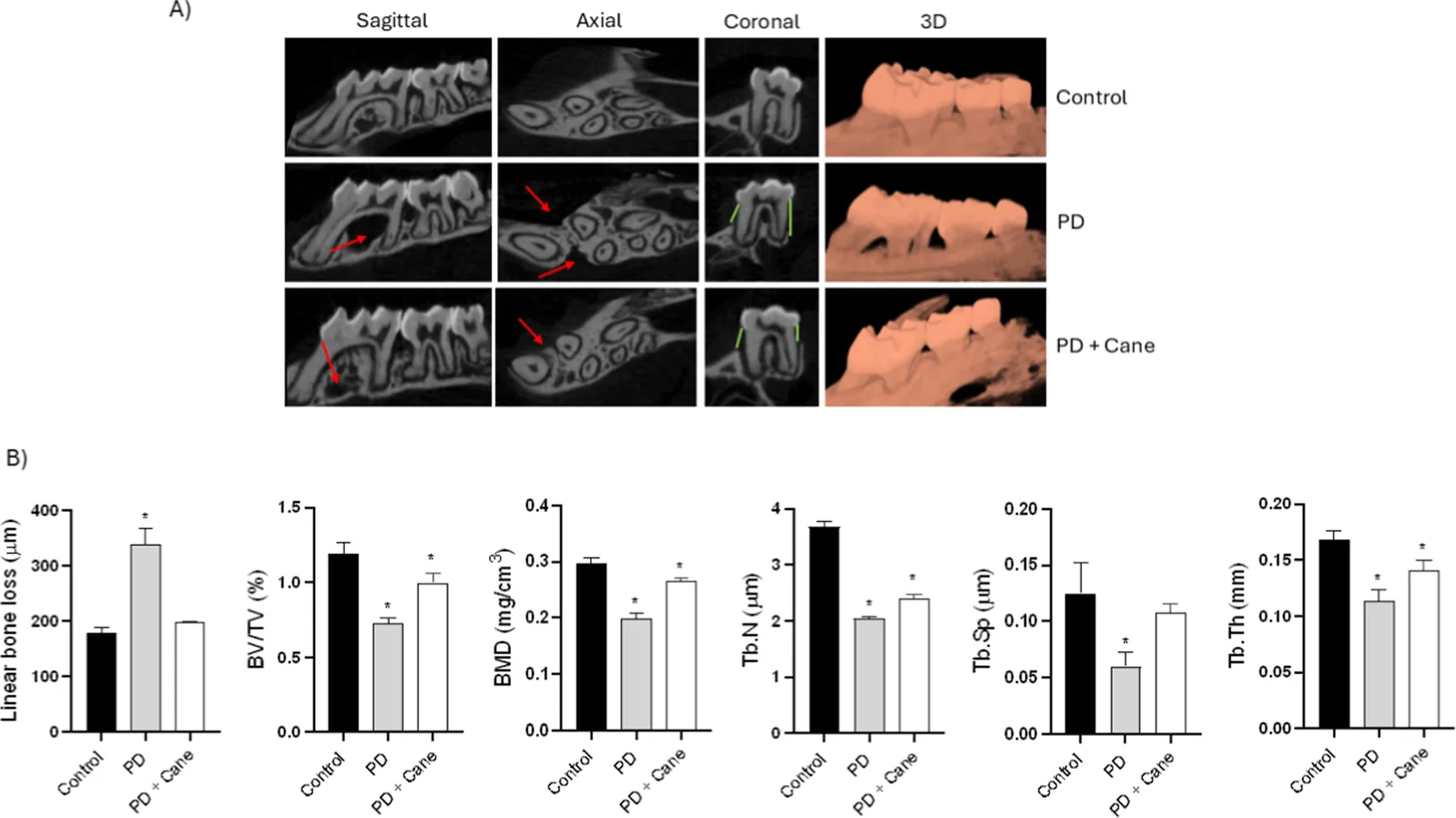
(A–B)—(A) Representative images of sagittal, axial, coronal and three-dimensional micro-CT views of maxillary molars samples from each experimental group. (B) Micro-CT analyses of linear bone loss, percentage of BV/TV, BMD and number, separation and thickness of trabecular bone. *Significant difference compared with all other groups. (ANOVA/Tukey, *p* < 0.05).

These histological features may reflect changes in periodontal tissue organization associated with a less inflammatory profile. In a previous in vitro study, CaneCPI-5 has been reported to stimulate cellular differentiation, migration and proliferation through modulation of cell-cycle regulators and cytoskeletal remodeling pathways.^20^ Such biological activities may contribute to tissue repopulation and repair processes, potentially helping to explain the increased fibroblast density and improved connective tissue organization observed in the periodontal tissues of treated animals. Extracellular matrix remodeling may represent another relevant mechanism. In addition, CaneCPI-5 enhances MMP-2 and MMP-9 activity during osteoblast differentiation,^20^ suggesting an active role in matrix turnover and tissue remodeling. Since controlled extracellular matrix remodeling is essential for both periodontal wound healing and mineralized tissue formation, this mechanism may have contributed to the more organized periodontal architecture observed in the treated animals.^31^ The convergence of in vitro and in vivo findings is consistent with a protective effect of CaneCPI-5 on both cellular behavior and periodontal tissue responses during inflammation, although the present data do not establish a direct causal relationship between the preservation of osteogenic activity and the attenuation of alveolar bone loss. From a translational perspective, local delivery may be a suitable approach for CaneCPI-5, given the localized nature of periodontal disease. Biomaterial-based systems, such as scaffolds, hydrogels, and membranes, could potentially be used to provide sustained and localized release of phytocystatins at periodontal defects. CaneCPI-5 could be investigated as an adjunct to established periodontal regenerative and host-modulation therapies rather than as a replacement for current approaches. Its optimal dose, delivery system, stability, and local and systemic safety remain to be established.

A limitation of the present study is that inflammatory mediators and signaling pathways were not directly evaluated. Therefore, although previous studies suggest anti-inflammatory effects of phytocystatins, the contribution of inflammatory modulation to the protective effects observed here remains speculative and should be investigated in future studies. While the precise molecular mechanisms underlying the effects of CaneCPI-5 remain to be fully elucidated, the present findings suggest that this phytocystatin may contribute to the maintenance of osteogenic activity and periodontal tissue preservation under inflammatory conditions. Current understanding of periodontal ligament cell biology indicates that inflammatory microenvironments can interfere with osteogenic processes through multiple regulatory pathways, and interventions capable of supporting pro-regenerative signaling may therefore be of clinical interest.

This study was conducted exclusively in male mice, which also represents a limitation regarding the generalizability of the findings. Experimental studies have demonstrated sex-related differences in periodontitis, including differences in alveolar bone loss and inflammatory responses in ligature-induced models of periodontal disease. In particular, female mice have been reported to exhibit greater periodontal bone loss and higher expression of pro-inflammatory cytokines than male mice, indicating that sex may influence the host response to periodontal challenge.^32^ Importantly, recent evidence indicates that biological sex may also influence the response to therapeutic modulation of periodontal inflammation, with pharmacological targeting of inflammatory pathways producing different protective effects in male and female mice.^33^ Therefore, because the protective effects of CaneCPI-5 occur within this inflammatory environment, sex-related differences in the magnitude and regulation of the host response could potentially influence its biological effects. However, whether the effects of CaneCPI-5 are sex-dependent remains unknown. Future studies including both male and female animals are warranted to determine whether the periodontal protective effects of CaneCPI-5 are consistent across sexes.

## Methods

### In Vitro Study

#### Cell Culture and Stimulation

An immortalized human periodontal ligament cell line (hPDLC) was cultured according to previously established protocols.^34,35^ Cells were cultured in Dulbecco’s Modified Eagle Medium (DMEM; Invitrogen, Karlsruhe, Germany) supplemented with 10% fetal bovine serum (FBS; Invitrogen), 100 U/mL penicillin, and 100 μg/mL streptomycin, and maintained at 37 °C in a humidified atmosphere containing 5% CO_2_. For gene expression analyses, hPDLCs were seeded at a density of 1 × 10^5^ cells per well in 6-well culture plates. For mineralization assays, cells were seeded at a density of 1 × 10^3^ cells per well in 24-well plates. Cells were treated with CaneCPI-5 at two different concentrations: 0.02 μg/μL (Cane 20) and 0.05 μg/μL (Cane 50). To mimic periodontal inflammatory conditions in vitro, hPDLCs were stimulated with the periodontopathogenic bacterium *F. alocis* (F.a.) (ATCC 35896), at a concentration previously established in our studies^36^ (MOI of 50:1, corresponding to 0.6 × 10^8^ CFU/mL). Bacterial strains were initially grown on Schaedler agar plates (Oxoid, Basingstoke, UK) for 48 h in an anaerobic environment. After cultivation, bacterial cells were collected and suspended in phosphate-buffered saline (PBS; Invitrogen, Thermo Fisher Scientific, Waltham, MA, USA). Complete bacterial killing was achieved by exposing the suspensions to two rounds of ultrasonication at 160 W for 15 min each, generating nonviable bacterial preparations for subsequent analyses. The use of nonviable bacterial preparations was intended to provide a controlled and reproducible bacterial stimulus while avoiding bacterial proliferation during the experimental period.

All experiments were performed in triplicate and repeated independently three times.

### Real-Time PCR

After 24 h of stimulation, cells were harvested for gene expression analysis. Total RNA was extracted using the RNeasy Mini Kit (Qiagen, Hilden, Germany) according to the manufacturer’s instructions, and RNA concentration was determined with a NanoDrop ND-2000 spectrophotometer (Thermo Fisher Scientific, Waltham, MA, USA). Subsequently, 500 ng of total RNA were reverse-transcribed into cDNA using the iScript Select cDNA Synthesis Kit (Bio-Rad Laboratories, Munich, Germany), following the manufacturer’s protocol. Quantitative real-time PCR (qPCR) was performed using a CFX96 thermal cycler (Bio-Rad Laboratories), SYBR Green master mix (QuantiFast SYBR Green PCR Kit, Qiagen), and specific human primers (QuantiTect Primer Assay, Qiagen). The expression levels of glyceraldehyde-3-phosphate dehydrogenase (GAPDH – ID: QT02504278), bone morphogenetic protein 2 (BMP2 – ID: QT00012544), osteopontin (SPP1 – ID: QT01008798), and collagen type I alpha 1 chain (COL1A1 – ID: QT00037793) were analyzed. Relative gene expression was calculated using the comparative threshold cycle method.

### Alizarin Red S Staining

Mineralized matrix formation was evaluated by Alizarin Red S staining to detect calcium-rich deposits. After 21 days of culture, cells were rinsed with phosphate-buffered saline and fixed in 70% ethanol. Subsequently, samples were incubated with a 2% Alizarin Red S solution (Sigma-Aldrich) for 15 min at room temperature, followed by washing to remove excess dye. For quantitative analysis, the bound stain was solubilized using a 10% cetylpyridinium chloride solution, and the absorbance was measured at 562 nm using a microplate reader (Asys-UVM 340, Biochrom). Mineralization was expressed as optical density values.

### In Vivo Study

#### Ligature-Induced Periodontitis Model

Thirty male C57BL/6J mice, 8 weeks old and weighing approximately 25 g, were used in this study. Animals were obtained from the institutional animal facility. All experimental procedures were performed following the protocols evaluated and approved by the Animal Ethics Committee of the School of Dentistry at Araraquara (protocol number 07/2022) and were conducted in accordance with the ARRIVE guidelines for the care and use of laboratory animals. Mice were housed under controlled environmental conditions (temperature 22 ± 2 °C, 12 h light/dark cycle) with ad libitum access to food and water. To ensure animal welfare, clinical monitoring was performed daily throughout the experimental period. Animals were evaluated for signs of pain or distress, and adequate food and water intake. Humane end points were predefined and included a loss of more than 20% of initial body weight, severe surgical-site infection or persistent lethargy. Animals meeting any of these criteria would have been euthanized before the planned end point to prevent unnecessary suffering. No animals met the predefined humane end point criteria or required early euthanasia during the study. Following an acclimatization period, animals were randomly allocated into three experimental groups (*n* = 10 per group): Control - no periodontitis induction and no treatment; PD: ligature-induced periodontitis treated with vehicle (PBS), and PD + Cane: ligature-induced periodontitis treated with CaneCPI-5 (0.8 μg/g/day). Experimental periodontitis was induced under general anesthesia by intraperitoneal administration of ketamine (80 mg/kg) and xylazine (10 mg/kg). Subsequently, sterile 5–0 nylon ligatures (Ethicon) were placed around the maxillary first molars bilaterally to promote biofilm accumulation and periodontal inflammation. Animals in the Control group were not subjected to ligature placement.

### CaneCPI-5 Administration

CaneCPI-5 or PBS (vehicle) was administered once daily by intraperitoneal injection throughout the 10 day experimental period. The dosage and route of administration were selected based on previous studies investigating the therapeutic effects of the recombinant phytocystatin CsinCPI-2 in murine models.^13,14^

### Euthanasia and Sample Processing

At the end of the experimental period, animals were euthanized by anesthetic overdose. One hemimaxilla from each animal was randomly selected and fixed in 4% paraformaldehyde for 48 h, then stored in 70% ethanol until microcomputed tomography (micro-CT) analysis. After micro-CT scanning, specimens were decalcified in 10% EDTA, processed for paraffin embedding, and sectioned in the sagittal plane. Serial sections (5 μm thick) were mounted on glass slides and stained with hematoxylin and eosin (H&E) for histometric and stereometric analyses.

### Micro-Computed Tomography (Micro-CT) Analysis

Maxillary samples were scanned using a microcomputed tomography system (SkyScan 1176, Bruker Corporation, Kontich, Belgium). Image reconstruction was performed using NRecon software (version 1.6.6.0; SkyScan, Bruker Corporation). Linear measurements were performed using Data Viewer software (version 1.4.4; SkyScan, Bruker Corporation), to determine the distance between the cementoenamel junction (CEJ) and the alveolar bone crest (ABC). Three-dimensional analyses were performed on axial sections using CTAn software (CT Analyzer) (version 1.12.0.0; SkyScan, Bruker Corporation). Bone volume (BV), total tissue volume (TV), and the BV/TV ratio were calculated. Trabecular bone microarchitecture parameters included trabecular number (Tb.N), trabecular separation (Tb.Sp), and trabecular thickness (Tb.Th). Grayscale thresholds for bone segmentation were determined using an automated thresholding algorithm (range 255–80). Bone mineral density (BMD) was calculated after calibration of CTAn software using two hydroxyapatite (CaOH) phantom rods with densities of 0.25 g/cm^3^ and 0.75 g/cm^3^.

### Histometric Analysis

For histometric evaluation, serial sagittal sections of 5 μm thickness, spaced at 100 μm intervals, were obtained and stained with H&E. Analyses were performed by a blinded and calibrated examiner. Measurements were carried out using a Leica DMLS light microscope (Leica; Reichert Diastar Products & Jung, Wetzlar, Germany) at 200× magnification. The linear distance from the cementoenamel junction (CEJ) to the alveolar bone crest (ABC) was measured, as well as the furcation region, which was defined as the area located 1000 μm apical to the furcation roof in the interradicular space of the maxillary first molars.

### Stereometric Analysis

Stereometric analysis was performed using the point-counting method to assess the relative abundance of the following tissue components: fibroblasts, inflammatory cells, blood vessels, and extracellular matrix. A bidimensional stereometric grid composed of 9 × 4 squares, covering a total area of 17,765 μm^2^, with each square measuring 30 μm, was constructed. The grid was created and overlaid onto digital images using Adobe Photoshop CS6 (Adobe Systems Incorporated, San Jose, CA, USA). Quantitative analysis was performed using ImageJ software (version 1.54d), and results were expressed as the percentage of points corresponding to each tissue component relative to the total number of points counted.

### Statistics

Sample size for the in vivo study was calculated using G*Power software (version 3.1.9.7), based on a one-way ANOVA and a previously published study.^37^ Alveolar bone loss was considered the primary outcome. The calculation indicated that a minimum of eight animals per group was required to detect the expected effect size with 80% statistical power (1 – β = 0.80) at an alpha level of 0.05 (α = 0.05). To account for a potential attrition rate of up to 20% due to unforeseen surgical or anesthetic complications, 10 animals per group were included. Animals were randomly allocated to the experimental groups, and each animal was considered the experimental unit.

Statistical analyses were performed using GraphPad Prism 9 (GraphPad Prism 9 Software, Inc., La Jolla, San Diego, CA, USA). The Shapiro–Wilk test was used to confirm the normal distribution of all data (*p* > 0.05). One-way analysis of variance (ANOVA) was followed by a post hoc Tukey’s test for multiple comparisons among groups (*p* ≤ 0.05).

## Conclusion

CaneCPI-5 preserved osteogenic activity in human periodontal ligament cells under basal and inflammatory conditions and was associated with reduced alveolar bone loss in experimental periodontitis. These findings suggest that CaneCPI-5 may support periodontal tissue stability in inflammatory environments. Further studies are required to elucidate its mechanisms and therapeutic potential.

## Data Availability

The data underlying this article will be shared on reasonable request to the corresponding author.

## Abbreviations

ABC: alveolar bone crest
BMD: bone mineral density
BMP2: bone morphogenetic protein 2
BV: bone volume
BV/TV: bone volume/total tissue volume
CEJ: cementoenamel junction
CFU: colony-forming units
COL1A1: collagen type I alpha 1 chain
DMEM: dulbecco’s modified eagle medium
EDTA: ethylenediaminetetraacetic acid
ERK: extracellular signal-regulated kinase
FBS: fetal bovine serum
GAPDH: glyceraldehyde-3-phosphate dehydrogenase
H&E: hematoxylin and eosin
hPDLCs: human periodontal ligament cells
micro-CT: microcomputed tomography
MMP-2: matrix metalloproteinase-2
MMP-9: matrix metalloproteinase-9
PBS: phosphate-buffered saline
PD: periodontitis
qPCR: quantitative polymerase chain reaction
RNA: ribonucleic acid
RT-qPCR: reverse transcription quantitative polymerase chain reaction
SPP1: secreted phosphoprotein 1
Tb.N: trabecular number
Tb.Sp: trabecular separation
Tb.Th: trabecular thickness
